# Survival Following Relapse in Children with Acute Myeloid Leukemia: A Report from AML-BFM and COG

**DOI:** 10.3390/cancers13102336

**Published:** 2021-05-12

**Authors:** Mareike Rasche, Martin Zimmermann, Emma Steidel, Todd Alonzo, Richard Aplenc, Jean-Pierre Bourquin, Heidrun Boztug, Todd Cooper, Alan S. Gamis, Robert B. Gerbing, Iveta Janotova, Jan-Henning Klusmann, Thomas Lehrnbecher, Nora Mühlegger, Nils v. Neuhoff, Naghmeh Niktoreh, Lucie Sramkova, Jan Stary, Katharina Waack, Christiane Walter, Ursula Creutzig, Michael Dworzak, Gertjan Kaspers, Edward Anders Kolb, Dirk Reinhardt

**Affiliations:** 1 Department of Pediatric Hematology-Oncology, Pediatrics III, University Hospital of Essen, 45147 Essen, Germany; emma.steidel@uk-essen.de (E.S.); nils.vonneuhoff@uk-essen.de (N.v.N.); naghmeh.niktoreh@uk-essen.de (N.N.); katharina.waack@uk-essen.de (K.W.); christiane.walter2@uk-essen.de (C.W.); dirk.reinhardt@uk-essen.de (D.R.); 2 Department of Pediatric Hematology and Oncology, Hannover Medical School, 30625 Hannover, Germany; zimmermann.martin@mh-hannover.de (M.Z.); ucreutzig@onlinehome.de (U.C.); 3 Department of Preventive Medicine, Keck School of Medicine, University of Southern California, Los Angeles, CA 90033, USA; talonzo@childrensoncologygroup.org; 4 Division of Oncology, The Children’s Hospital of Philadelphia, Philadelphia, PA 19104, USA; APLENC@email.chop.edu; 5 Division of Pediatric Hematology/Oncology, University Children’s Hospital Zurich, CH-8032 Zurich, Switzerland; Jean-Pierre.Bourquin@kispi.uzh.ch; 6 St Anna Children’s Hospital and Children’s Cancer Research Institute, Department of Pediatrics, Medical University of Vienna, 1090 Vienna, Austria; heidrun.boztug@stanna.at (H.B.); nora.muehlegger@ccri.at (N.M.); michael.dworzak@stanna.at (M.D.); 7 Seattle Children’s Hospital, Seattle, WA 98105, USA; Todd.Cooper@seattlechildrens.org; 8 Children’s Mercy Hospitals and Clinics, Kansas City, MO 64108, USA; agamis@cmh.edu; 9 Children’s Oncology Group, Monrovia, CA 91016, USA; rgerbing@childrensoncologygroup.org; 10 Department of Pediatric Hematology and Oncology, 2nd Faculty of Medicine, Charles University and University Hospital Motol, 150 06 Prague, Czech Republic; Iveta.Janotova@fnmotol.cz (I.J.); Lucie.Sramkova@fnmotol.cz (L.S.); jan.stary@lfmotol.cuni.cz (J.S.); 11 Clinic for Pediatrics 1, Martin Luther University Halle-Wittenberg, 06108 Halle, Germany; Jan-Henning.Klusmann@uk-halle.de; 12 Division for Pediatric Hematology and Oncology, Hospital for Children and Adolescents, University Hospital, Goethe University Frankfurt am Main, 60590 Frankfurt, Germany; Thomas.Lehrnbecher@kgu.de; 13 Princess Máxima Center for Pediatric Oncology, 3584 CS Utrecht, The Netherlands; gjl.kaspers@vumc.nl; 14 Pediatric Oncology, Emma Children’s Hospital, Amsterdam UMC, Vrije Universiteit Amsterdam, 1105 AZ Amsterdam, The Netherlands; 15 Nemours/Alfred I. du Pont Hospital for Children, Wilmington, DE 19803, USA; Edward.Kolb@nemours.org

**Keywords:** acute myeloid leukemia, relapse, childhood acute myeloid leukemia, pediatric, salvage therapy

## Abstract

**Simple Summary:**

Acute myeloid leukemia in children remains a difficult disease to cure despite intensive therapies that push the limits of tolerability. Though the intent of initial therapy should be the prevention of relapse, about 30% of all patients experience a relapse. Hence, relapse therapy remains critically important for survival. This retrospective analysis of two large international study groups (COG and BFM) was undertaken to describe the current survival, response rates and clinical features that predict outcomes. We demonstrate that children with relapsed AML may be cured with cytotoxic therapy followed by HSCT. High-risk features at initial diagnosis and early relapse remain prognostic for post-relapse survival. Current response criteria are not aligned with the standards of care for children, nor are the count recovery thresholds meaningful for prognosis in children with relapsed AML. Our data provide a new baseline for future treatment planning and will allow an updated stratification in upcoming studies.

**Abstract:**

Post-relapse therapy remains critical for survival in children with acute myeloid leukemia (AML). We evaluated survival, response and prognostic variables following relapse in independent cooperative group studies conducted by COG and the population-based AML-BFM study group. BFM included 197 patients who relapsed after closure of the last I-BFM relapse trial until 2017, while COG included 852 patients who relapsed on the last Phase 3 trials (AAML0531, AAML1031). Overall survival at 5 years (OS) was 42 ± 4% (BFM) and 35 ± 2% (COG). Initial high-risk features (BFM 32 ± 6%, COG 26 ± 4%) and short time to relapse (BFM 29 ± 4%, COG 25 ± 2%) predicted diminished survival. In the BFM dataset, there was no difference in OS for patients who had a complete remission with full hematopoietic recovery (CR) following post-relapse re-induction compared to those with partial neutrophil and platelet recovery (CRp and CRi) only (52 ± 7% vs. 63 ± 10%, *p* = 0.39). Among 90 patients alive at last follow-up, 87 had received a post-relapse hematopoietic stem cell transplant (HSCT). OS for patients with post-relapse HSCT was 54 ± 4%. In conclusion, initial high-risk features and early relapse remain prognostic. Response assessment with full hematopoietic recovery following initial relapse therapy does not predict survival. These data indicate the need for post-relapse risk stratification in future studies of relapse therapies.

## 1. Introduction

The prognosis of children with acute myeloid leukemia (AML) has improved in recent decades, with current overall survival rates of approximately 70% [1,2,3,4,5,6]. Most international study groups currently utilize treatment regimens that include four to five courses of intensive myelosuppressive chemotherapy or intensive chemotherapy followed by hematopoietic stem cell transplantation (HSCT) for high-risk (HR) patients [7,8]. Despite intensive regimens that push the limits of tolerance, relapse rates as high as 30% have improved little over the past twenty years [7]. Post-relapse therapy remains critical for survival in childhood AML [5,9,10,11].

There has been considerable improvement in overall probability of survival (pOS) for patients in first relapse since 1987, improving from a 5-year-pOS of 21–23% in the 1980s and 1990s [11,12,13] to 36–39% in patients’ relapse prior to 2014 [9,14,15,16]. Studies consistently demonstrate that duration of first remission (CR1), age at relapse (less than ten years), favorable cytogenetics such as core binding factor (CBF) AML and good treatment response after re-induction therapy predict a more favorable outcome [9,10,11,12,13,14,17,18,19,20,21].

In this retrospective analysis of children with AML in first relapse, we report post-relapse response, survival estimates and prognostic variables from the BFM registry and recent COG Phase III trials. These data represent the largest available relapse AML datasets analyzed for post-relapse survival.

## 2. Materials and Methods

### 2.1. Patients

Datasets included patients treated in the United States, Canada, Australia, New Zealand, Germany, Austria, Czech Republic and Switzerland.

**BFM relapse cohort**. The AML-BFM registry includes patients diagnosed with de novo AML between 2004 and 2017, who have been enrolled in the multicenter population-based AML-BFM study group trials and registries (AML-BFM study 2004—ClinicalTrials.gov Identifier: NCT00111345, AML-BFM registry 2012 and AML-BFM study 2012—EudraCT 2013-000018-39). Figure S1 provides a consort diagram accounting for all BFM patients since 2004.

Included are children with documented first relapse between April 2009 and December 2017 (*n* = 197). The primary analysis is limited to those patients who experienced a relapse after closure of the last I-BFM Relapsed trial Acute Myeloid Leukemia 2001/01. However, the previously described 513 patients enrolled before 04/2009 on Relapsed Acute Myeloid Leukemia 2001/01 [9] are included for comparison.

**COG relapse cohort**. All patients enrolled on COG AAML0531 (NCT00372593 [22]) and AAML1031 (NCT01371981 [6]) who relapsed are included (*n* = 852: AAML0531 *n* = 358, and AAML1031 *n* = 494). Eligibility, therapy and results for these trials have been previously reported [6,22]. Only patients with DS-AML enrolled on AAML0531 are excluded from this post-relapse analysis. Residual disease (RD) was evaluated by central flow cytometry as previously described [23,24] at the end of one cycle of initial induction therapy in 765 patients (90%). Disease characteristics are well-characterized at diagnosis, but post-relapse data collection in the COG cohort is limited to survival.

National ethics committees and institutional review boards approved all studies and patients or guardians provided written informed consent. The retrospective analysis and all included studies were performed in accordance with the Declaration of Helsinki.

### 2.2. Definitions and Cohorts

Five-year estimate of the probability of post-relapse overall survival (pOS) was defined as time from date of first relapse to date of last follow-up or death from any cause. The 5-year estimate of event-free survival (pEFS) was defined as time from diagnosis at first relapse to the next event (second relapse, death of any cause, failure to achieve second remission or secondary malignancy) or date of last follow-up. Failure to achieve second remission was considered an event on day 0. Additional definitions are listed in Table S1.

A detailed response evaluation is included for a subset of uniformly treated BFM patients. The remission status data after first re-induction were derived from a bone marrow aspiration performed prior to a second re-induction. Second re-induction commenced at the discretion of the treating investigator and did not require hematopoietic recovery [25,26].

### 2.3. Statistical Analyses

Statistical analyses were performed with SAS (SAS Institute version 9.4, Cary, NC, USA). Median follow-up after diagnosis of first relapse was 4.2 years (0.3–10.3 years) in the AML-BFM cohort and 4.6 years (0–11.1 years) for the COG cohort. The Kaplan–Meier method was applied to estimate probabilities of survival. EFS and OS were compared with the log-rank test. Cumulative incidence functions of early death or relapse were constructed according to Kalbfleisch and Prentice. The Cox proportional hazards model was used for multivariate analysis of outcomes. We selected risk factors that have been significant in univariate analysis in one or both study groups for the multivariate analysis: risk group at initial diagnosis (inv(16)(p13.1q22), t(8;21)(q22;q22.1), high-risk), nonresponse at initial disease, time to relapse, date of relapse diagnosis and age at relapse. Proportions were compared between groups using the Chi-square or Fisher’s exact test. *p* values < 0.05 were considered significant. Living patients were censored at date of last follow-up. Data were frozen at 03/27/2020 (BFM) and 03/31/2020 (COG).

## 3. Results

### 3.1. Patient Characteristics and Previous Treatment

**BFM cohort**—Using a combined cytomolecular and response-guided risk stratification at initial disease (Table S1), 68 patients (38%) were classified as high-risk, 81 (45%) were intermediate and 31 (17%) standard risk (unclassified, *n* = 17). Forty-six percent of patients experienced a first relapse within one year of diagnosis. Fourteen percent of the patients (*n* = 28) had an HSCT prior to relapse. More details are shown in Table S2.

**COG cohort**—All relapsed patients were reclassified using the AAML1031 risk stratification (Table S1). Across the two studies, 608 patients (72%) were classified as low-risk at initial diagnosis and 237 (28%) high-risk (no data, *n* = 7, 1%). Among the high-risk patients, 194 (82%) had residual disease detected by flow cytometry at the end of one cycle of initial induction chemotherapy. Early relapse (within 1 year of diagnosis) occurred in 500 patients (59%), while 352 patients relapsed more than 1 year from diagnosis (41%). One hundred seventeen patients (14%) received an HSCT prior to relapse, 670 (77%) had no prior HSCT and 65 (8%) have insufficient data. Additional patient characteristics are shown in Table S3.

### 3.2. Post-Relapse Therapy

**BFM cohort**—After closure of the last relapse trial in 2009 [9], BFM guidelines recommended fludarabine, cytarabine, liposomal daunorubicin (DNX-FLA) followed by FLA and HSCT. Subsequently, 81% (*n* = 156) of all patients received the treatment with DNX-FLA(G) with or without second FLA. Fourteen percent of the patients had an alternate second cycle, primarily due to nonresponse in relapse and often including Gemtuzumab Ozogamicin (GO) or Clofarabine. Four percent of the patients received palliative care only (Table 1). Most patients who received an intensive re-induction treatment proceeded to transplant (81% of all patients and 87% of patients receiving DNX-FLA). Ten percent (*n* = 15) of the transplanted patients had a prior HSCT (Table 1). Among the 90 patients alive at last follow-up, 87 had a post-relapse HSCT, two are unknown and one patient alive did not receive an HSCT. Of note, the rate of HSCT following relapse increased over time. Sixty-nine percent of the patients included in the first time period of the previous relapse trial AML 2001/01 were able to proceed to HSCT, while 82% percent of the patients had an HSCT in the recent time interval (p(chi) = 0.0286; Table S4).

### 3.3. Survival and Prognostic Factors at First Relapse

**Figure 1:**
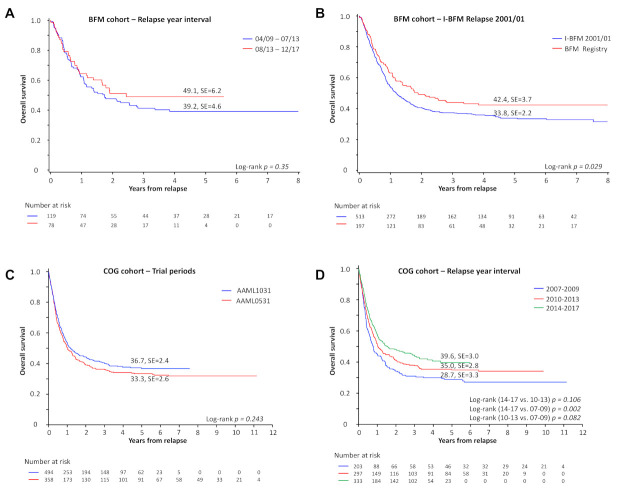
Overall survival at first relapse. (**A**) Five-year overall survival in patients with pediatric AML with diagnosed first relapse from 04/2009 until 07/2013 vs. 08/2013—12/2017. (**B**) Five-year overall survival in patients with pediatric AML with first relapse enrolled in the BFM registry compared to patients with first relapse enrolled in the previous I-BFM Relapse 2001/01 trial. (**C**) Five-year overall survival following relapse of patients enrolled on COG AAML0531 and COG AAML1031. (**D**) Five-year overall survival for first relapse patients in the COG cohort by year. Group 2018 until 2019 is excluded.

**Overall survival.** The 5-year pOS for patients in the AML-BFM studies is 42 ± 4% (Table S5). For patients relapsing between 08/2013 and 12/2017, 5-year pOS was 49 ± 6% (Figure 1A). Time to death was comparable between both intervals (08/2013 until 12/2017 vs. 04/2009 until 07/2013: *p* = 0.2263). The 90th percentile of time to death from 04/2009 to 07/2013 was 2.14 years. Sixty-four percent of surviving patients who relapsed between 08/2013 and 12/2017 had more than 2.14 years of follow-up. Median follow-up of patients from 04/2009 to 07/2013 was 4.7 years and 2.8 years from 08/2013 to 12/2017.

With a median follow-up of 4.9 years, the pOS of all patients enrolled on the Acute Myeloid Leukemia 2001/01 trial [9] is 34 ± 2%, compared to a pOS of 42 ± 4% (median follow-up 4.3 years) in the current BFM dataset (Figure 1B, *p* = 0.029). There is a trend towards improved survival over time (Figure S2A). However, when comparing only patients who received DNX-FLA or DNX-FLA and HSCT, survival was comparable between the two treatment periods (Figure S2B, pOS 44 ± 4% vs. 37 ± 4%, *p* = 0.16) and over time (Figure S2C).

The survival of the 157 patients who received an HSCT following relapse was 52 ± 4% (Figure S3A). Only one long-term surviving patient is alive without a post-relapse HSCT. This patient received Sorafenib and donor lymphocyte infusions (DLIs).

Among 157 patients who underwent HSCT following first relapse, 15 patients had a prior HSCT in first remission (93% for HR disease at initial diagnosis). The pOS was 28 ± 13% (*n* = 15) vs. 55 ± 4% in patients with first HSCT (*n* = 142; p(KM test) = 0.06; Figure S3B). When limiting the same analysis to just the HR patients, the pOS was 31 ± 14% for patients receiving a second transplant at relapse (*n* = 14) compared to a pOS 47 ± 9% for patients receiving their first transplant following relapse (*n* = 34, p(KM test) = 0.75; Figure S3C).

The 5-year pOS for the entire COG cohort is 35 ± 2%. For patients relapsing after treatment on AAML0531, the 5-year pOS is 33 ± 3% and for AAML1031 the 5-year pOS is 37% ± 2% (Figure 1C). For patients relapsing between 2013 and 2017, 5-year pOS was 40% ± 3% (Figure 1D). The 90th percentile of time to death from 04/2009 to 07/2013 was 2.0 years. Eighty-five percent of surviving patients who relapsed between 08/2013 and 12/2017 had more than 2.0 years of follow-up. Median follow-up of patients from 04/2009 to 07/2013 was 6.3 years and 3.6 years from 08/2013 to 12/2017.

**Figure 2:**
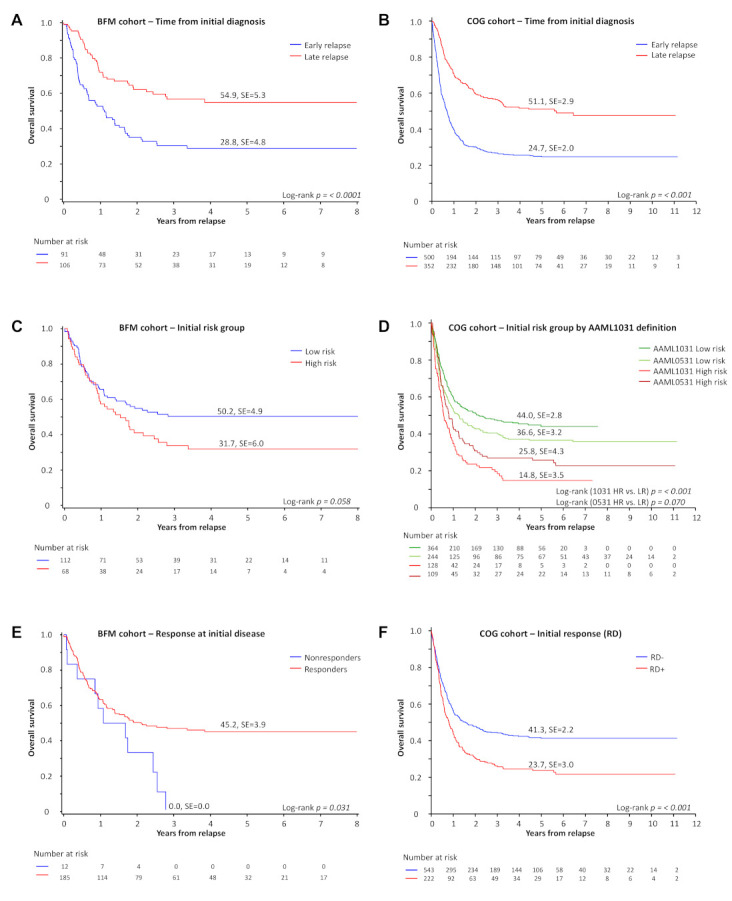
Prognostic factors for post-relapse survival. (**A**) Five-year overall survival of patients with early or late relapse defined as relapse within or after one year of initial diagnosis (BFM). (**B**) Five-year overall survival of patients with early or late relapse (COG). (**C**) Five-year overall survival in patients with first relapse based on the genetic risk profile of the initial diagnosis (BFM). (**D**) Five-year overall survival from relapse for AAML1031 and AAML0531 patients based on a retrospective classification by AAML1031 risk group definition (Table S1). (**E**) Five-year overall survival of patients based on the initial response to induction chemotherapy of the initial disease (BFM). Abbreviations: HR, high-risk. (**F**) Five-year overall survival according to residual disease detection at the end of one cycle of induction following initial diagnosis and treatment (COG).

**Time to relapse**. Survival of BFM patients experiencing early relapse within one year from initial diagnosis was significantly reduced (pOS 29 ± 5%, *n* = 91 vs. 55 ± 5%, *n* = 106; *p* < 0.0001; Figure 2A). Patients with a time to relapse that was less than 6 months showed a comparable outcome to patients relapsing within 6–12 months (*n* = 19, pOS 37 ± 11% vs. *n* = 72, pOS 27 ± 5%; *p* = 0.55; data not shown). Although the treatment year was not significant in multivariable analysis, there is a trend towards improvement in survival that is limited to patients with a late relapse (Figure S4A,B). In the COG cohort, the 5-year pOS was 25 ± 2% for patients relapsing within one year from initial diagnosis (*n* = 352) and 51 ± 3% for patients relapsing later (*n* = 500, *p* < 0.001; Figure 2B).

**Figure 3:**
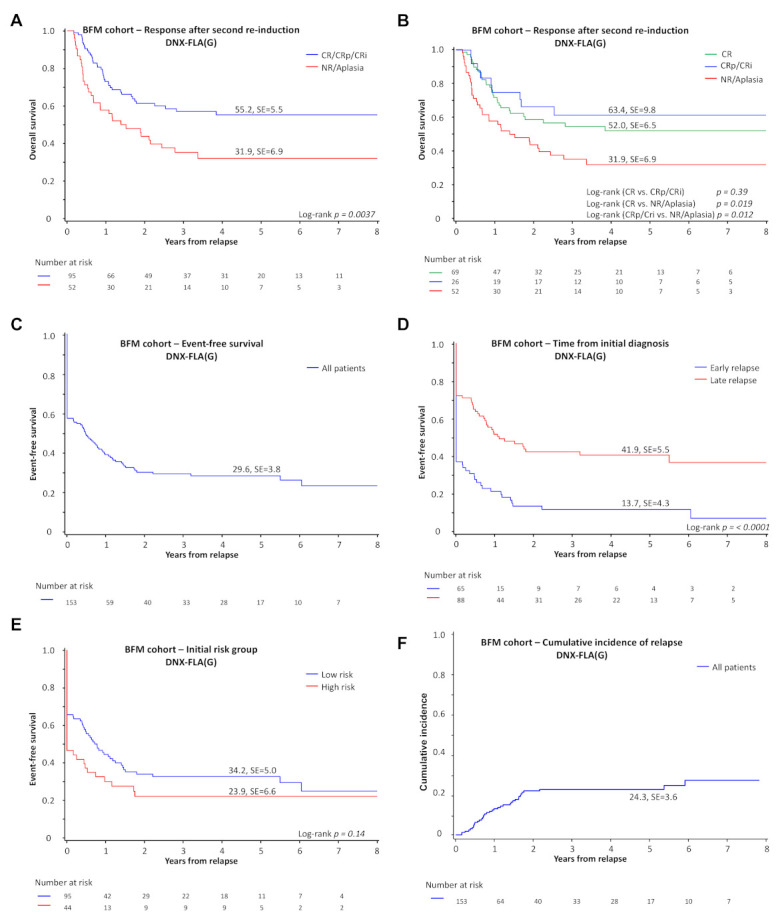
Response and EFS at first relapse. (**A**) Five-year overall survival in patients with pediatric AML with first relapse based on the response to DNX-FLA(G) +/− FLA(G) comparing complete remission with complete (CR) and partial regeneration (CRp) and CRi vs. nonresponse and aplasia. (**B**) Five-year overall survival in patients with pediatric AML with first relapse based on the detailed response to DNX-FLA(G) +/− FLA(G). (**C**) Five-year event-free survival in all patients receiving DNX-FLA(G) +/− FLA(G) after first relapse. (**D**) Five-year event-free survival of patients with early or late relapse defined as relapse within or after one year of initial diagnosis. (**E**) Five-year event-free survival in patients with first relapse based on the risk profile of the initial diagnosis. (**F**) Five-year cumulative incidence of a second relapse in all patients receiving DNX-FLA(G) +/− FLA(G). The competing event is death. Abbreviations: HR, high-risk. DNX-FLA(G), liposomal daunorubicin, fludarabine, cytarabine with or without granulocyte-colony-stimulating factor. CR, complete remission; CRp, complete remission with partial regeneration; Cri, complete remission with incomplete recovery. See Table S1 for definitions.

**Initial risk classification**. The pOS was 31 ± 6% in patients initially characterized as high-risk in the BFM cohort (Table S1) when compared to non-HR patients (50 ± 5%, p(LR) = 0.058) (Figure 2C). When COG AAML0531 patients are reclassified using the AAML1031 risk assignment definitions (Table S1), the 5-year pOS for initial HR patients was 26 ± 4% compared to 37 ± 3% for LR patients (*p* = 0.07; Figure 2D). Patients enrolled on AAML1031 and classified as HR had a 5-year pOS of 15 ± 4% compared to 44 ± 3% for patients initially classified as LR (*p* < 0.001; Figure 3D).

**Response to initial induction**. Response to induction therapy was evaluated differently for COG and BFM data sets; however, poor response in both groups predicts poor survival. Nonresponse to therapy for initial disease (≥10% blasts after first or ≥5% after second induction) in the BFM cohort translated into a dismal prognosis after relapse compared to those who responded well at initial diagnosis (pOS 0 ± 0%, *n* = 12 vs. pOS 45 ± 4%, *n* = 185; *p* = 0.031; Figure 2E and Table S5). In the COG cohort, 765 of the 852 (90%) were evaluated for residual disease (RD) by central flow cytometry at the end of one cycle of initial induction therapy. In total, 222 (29%) had RD. The 5-year pOS following relapse for patients who were RD-positive at the end of initial induction was 24 ± 3% (*n* = 222) and 41 ± 2% for those who were RD-negative (*n* = 543, *p* < 0.001) (Figure 2F).

**Univariable and multivariable risk analysis.** Univariable and multivariable Cox analyses from relapse are shown (Table 2).

By multivariable analysis in the BFM cohort, initial nonresponse at diagnosis (pOS: hazard ratio 1.80, 95% CI 1.18–2.75, p(chi) = 0.007) and an early relapse (pOS: hazard ratio 1.94, 95% CI 1.22–3.07, p(chi) = 0.005) independently predict outcome following relapse for all patients. The hazard ratio for high-risk criteria in the BFM cohort was 1.51 (95% CI 0.97 to 2.33, *p* = 0.065). In the COG cohort, multivariable analysis identified early relapse (hazard ratio 2.17, 95% CI 1.78 to 2.65) and high-risk group assignment (hazard ratio 1.50, 95% CI 1.23 to 1.81) to be associated with inferior pOS.

**Response and event-free survival after first relapse (BFM cohort).**

Within the BFM dataset, age, gender, white blood cell count or initial de novo treatment protocols were similar between the DNX-FLA group (*n* = 156). The remaining group had a higher proportion of patients with high-risk disease, nonresponse to initial therapy and early relapse (Table S2), several of whom received only palliative therapy at relapse.

Among the 156 patients who received DNX-FLA, 153 were evaluable for response (3 were excluded for insufficient data). After up to 2 induction courses, 69 patients (57%) achieved a CR, 20 a CRp (13%), 6 (4%) a CRi and 52 (34%) no response or aplasia. Six patients (4%) had an early death before response assessment. The pOS was superior (55 ± 6%, vs. 32 ± 7%; *p* = 0.0037; Figure 3A) for patients with a CR/CRp/CRi compared to patients with no response. Patients with a CRp or CRi (pOS 63 ± 10%) had a comparable overall survival to those with a CR (pOS 52 ± 7%) (*p* = 0.39; Figure 3B). The detailed analysis shows that patients with ≥5% leukemic blasts after second re-induction had the lowest survival with a pOS of 27 ± 9% (*n* = 32, Figure S5). The 5-year pEFS for this cohort of 153 patients was 30 ± 4% and was comparable in patients with CR and CRp (Figure 3C, CR: pEFS 50 ± 6%, *n* = 69, vs. CRp: pEFS 50 ± 11%, *n* = 20). The cumulative incidence of death before day 56 of relapse therapy was 4 ± 2% (Table S2). Of note, short time to relapse was associated with a reduced 5-year pEFS, while initial risk stratification did not reach significance (Figure 3D,E). The cumulative incidence of relapse in all patients (also including patients with initial nonresponse) was 24 ± 4% (Figure 3F).

## 4. Discussion

Survival data from pediatric patients treated within the AML-BFM protocols between 2004 and 2017 and COG Phase 3 trials between 2006 and 2018 were analyzed retrospectively. The 5-year pOS for 197 BFM relapse patients was 42 ± 4% and the 852 COG patients 35 ± 2%. Among the 156 BFM patients who received DNX-FLA following relapse, the 5-year pOS was 44%. When compared to an analysis of patients treated on the AML 2001/01 trial (5-year-pOS of 34%) [9], there is a trend towards improved survival over time, as well as increasing rates of post-relapse HSCT. This increase of patients proceeding to HSCT may account for improvements in survival. Since re-induction therapy has not improved, it is most likely that more children are receiving HSCT at relapse because of improved supportive care and donor availability.

Limitations within this retrospective review include the non-compulsory treatment schedule in the BFM dataset and the missing post-relapse treatment and response data in the COG dataset. Nonetheless, the results of our large retrospective analysis have important implications for future treatment planning. Previously published prognostic factors following a first relapse include time to relapse, treatment response at relapse, initial cytogenetics and HSCT in CR1 [9,10,11,12,13,14,17,18,19,20]. In the current study, multivariable analysis identified relapse within a year of diagnosis in both datasets and initial high-risk disease classification in the COG datasets are predictive of poor overall survival. Importantly, high-risk assignment in the COG cohort includes patients with detectable RD by flow cytometry at the end of initial induction therapy.

In the BFM registry, 156 patients (80%) received DNX-FLA at the discretion of the treating investigator. This homogeneously treated population permitted a retrospective analysis of response. Nonresponse rates after relapse re-induction therapy (21%) were higher at relapse than nonresponse rates reported in de novo AML studies (range of 3–18%) [1,2,3,4,5,6,27,28]. The outcome of children with no response after relapse (≥5% leukemic blasts after second cycle of re-induction) was poor but significant (5-year pOS of 27%), suggesting that HSCT has a role post-relapse even in the absence of a complete remission. Sixty-one percent of patients achieved a CR, CRp or CRi after up to two cycles or re-induction chemotherapy with DNX-FLA +/− FLA. Traditionally, the International Working Group (IWG) criteria for response require an absolute neutrophil count of 1 × 10^9^/L and platelets of 100 × 10^9^/L for a CR. These criteria imply that the absence of count recovery is prognostic. While retrospective studies in adult patients with de novo AML have indicated that outcomes are superior for patients with response and full hematologic recovery compared to those with Cri/CRp [29,30], such a claim has never been validated in children with AML. Within the BFM cohort, absence of full hematopoietic recovery following standard relapse re-induction therapy does not predict survival in children at relapse (Figure 3B). This is comparable to what is observed in a recent study with adult patients following a first relapse or refractory disease. Overall survival was similar in patients who achieved CR with full hematologic recovery vs. those with incomplete hematologic recovery [31].

The Cheson criteria, introduced in 1990 to assess response in adult de novo AML, should be reconsidered as the standard for response evaluation for children with AML [25,26,30], and perhaps adults with AML as well (as reviewed in Bloomfield et al. [32]). In the AML-BFM and COG studies, it is common to continue intensification of treatment without waiting for full hematopoietic recovery [6,8,22]. Our data again confirm that clinicians prioritize maintaining therapy intensification rather than waiting for a hematopoietic regeneration. Continuation of treatment without count recovery has produced favorable outcomes in North American and European trials for children with newly diagnosed AML [1,33]. The IWG/Cheson criteria are likely to underestimate response in children as they are not aligned with the standards of care for children, nor are the count recovery thresholds to achieve a CR meaningful in children with relapsed AML. It will be important in future studies of novel relapse therapies for children that the defined response criteria reflect standard treatment strategies specifically used for children.

## 5. Conclusions

The findings of our large retrospective analysis have important implications for future trials. Within this international cohort we confirmed that initial risk stratification and time to relapse are prognostic for post-relapse survival. We also declare, for the first time in children, that lack of a full hematopoietic regeneration to the thresholds required by the IWG response criteria is neither necessary for, nor predictive of, survival at first relapse in children. AML in children remains a difficult disease to cure despite intensive therapies that push the limits of tolerability. Though the intent of any initial therapy should be the prevention of relapse, nearly half of all relapse patients will still survive. Refinements in post-relapse care continue to show benefit in overall survival across cooperative groups. As we continue to evaluate innovative therapies to improve survival without adding cumulative short- and long-term toxicities, it is important to reevaluate poor risk features that predict survival at relapse and appropriate definitions for response in children.

## Figures and Tables

**Table 1:** Treatment and Response (BFM cohort).

		Patients (%)
First Relapse Treatment of Pediatric AML		*n* = 197
**Chemotherapy**	Re-Induction with DNX-FLA(G) +/−FLA (G)	156 (81%)
	Re-Induction with FLA(G) +/−FLA (G)	5 (3%)
	Re-Induction with Ida-FLA +/− FLA	3 (2%)
	Re-Induction with FLA + others +/− FLA	3 (2%)
	Re-Induction with a Clofarabine-containing regimen	8 (4%)
	Others (e.g., Gemtuzumab Ozagamicin, Sorafenib, intrathecal treatment only)	10 (5%)
	Palliative care	8 (4%)
	Unknown	4
**HSCT**	No HSCT	37 (19%)
	HSCT	157 (81%)
	First HSCT	142 (90%)
	Second HSCT	15 (10%)
	Unknown	3
**First relapse response and HSCT after DNX-FLA +/−FLA**		*n* = 156
**HSCT**	No HSCT	20 (13%)
	HSCT	134 (87%)
	First HSCT	123 (92%)
	Second HSCT	11 (8%)
	Unknown	2
**Response after 2 induction cycles**	CR	69 (45%)
	CRp	20 (13%)
	Cri	6 (4%)
	Aplasia	20 (13%)
	NR	32 (21%)
	Early death before CR evaluation	7 (5%)
	Unknown	2
**Early Treatment Response**	Death before evaluable BM	4 (3%)
	BM after first induction not available or not applicable	12 (8%)
	Evaluable BM after first induction	140 (90%)
	>20% leukemic blasts in the BM after first induction	18 (13%)
	≤20% leukemic blasts in the BM after first induction	122 (87%)

Table legend: For categories including patients with unknown status, percentages are calculated without “unknown”. Abbreviations: CR, complete remission; CRp, complete remission with partial regeneration; Cri, complete remission with incomplete recovery; DNX, liposomal daunorubicin; FLA(G), fludarabine, cytarabine with or without granulocyte colony-stimulating factor; HSCT, hematopoietic stem cell transplantation; NR, nonresponse. See Table S1 for definitions.

**Table 2:** Analysis of risk factors.

	BFM				COG			
Criteria	pOS				pOS			
	n (all pts.)	HR	95% CI	p (Chi)	n (all pts.)	HR	95% CI	p (Chi)
**Univariable analysis**								
Time from initial diagnosis <1 year	91 (197)	2.24	1.52–3.30	<0.001	500 (852)	2.31	1.92–2.79	<0.001
Age at relapse <2 years	36 (197)	1.00	0.53–1.87	0.998	116 (852)	1.41	1.09–1.83	0.009
Age at relapse 2–9 years	67 (197)	0.88	0.57–1.36	0.558	316 (852)	0.93	0.76–1.14	0.497
Age at relapse 10–13 years	39 (197)	0.68	0.36–1.27	0.228	124 (852)	0.93	0.71–1.22	0.601
Age at relapse >13 years	55 (197)	1			296 (852)	1		
inv(16)(p13.1q22)	7 (190)	0.83	0.26–2.62	0.751	72 (841)	0.32	0.21–0.49	<0.001
t(8;21)(q22;q22.1)	20 (192)	0.62	0.30–1.27	0.188	71 (841)	0.66	0.47–0.93	0.018
Nonresponse at initial disease	12 (197)	2.04	1.39–2.99	<0.001	--	--	--	--
RD at EOI of initial disease	--	--	--	--	222 (765)	1.55	1.28–1.88	<0.001
High-risk group *	68 (180)	1.47	0.98–2.19	0.060	237 (845)	1.57	1.33–1.85	<0.001
Poor response (> 20% leukemic blasts) after first re-induction	18 (140)	1.74	0.95–3.18	0.071	--	--	--	--
Relapse year interval 2007–2009	--	--	--	--	203 (852)	1.43	1.15–1.78	0.001
Relapse year interval 2010–2013 †	119 (197)	1.21	0.81–1.81	0.350	297 (852)	1.18	0.96–1.45	0.112
Relapse year interval 2014–2017 ‡	78 (197)	1			333 (852)	1		
Relapse year interval 2018–2019	--	--	--	--	19 (852)	1.26	0.64–2.45	0.507
**Multivariable analysis**								
Time from initial diagnosis < 1 year	81 (177)	1.95	1.23–3.09	0.005	493 (839)	2.17	1.78–2.65	<0.001
Age at relapse < 2 years	17 (177)	0.52	0.24–1.12	0.095	114 (839)	0.96	0.73–1.27	0.768
Age at relapse 2–9 years	68 (177)	0.76	0.47–1.22	0.256	311 (839)	0.79	0.64–0.98	0.029
Age at relapse 10–13 years	28 (177)	0.67	0.35–1.28	0.223	122 (839)	0.78	0.59–1.02	0.073
Age at relapse >13 years	64 (177)	1			292 (839)	1		
inv(16)(p13.1q22)	7 (177)	1.26	0.37–4.24	0.713	72 (839)	0.36	0.23–0.56	<0.001
t(8;21)(q22;q22.1)	20 (177)	0.96	0.44–2.11	0.922	71 (839)	0.73	0.51–1.03	0.075
Nonresponse at initial disease	11 (177)	1.80	1.18–2.76	0.006	--	--	--	--
High-risk group *	66 (177)	1.51	0.97–2.33	0.065	237 (839)	1.50	1.23–1.81	<0.001
Relapse year interval 2007–2009	--	--	--	--	200 (839)	1.22	0.97–1.52	0.090
Relapse year interval 2010–2013 †	103 (177)	1.13	0.73–1.74	0.586	294 (839)	1.10	0.89–1.35	0.376
Relapse year interval 2014–2017 ‡	74 (177)	1			326 (839)	1		
Relapse year interval 2018–2019	--	--	--	--	19 (839)	1.42	0.72–2.80	0.311

Table legend: Abbreviations: Chi, Chi-square test; CI, confidence interval; EOI, end of induction; HR, hazard ratio; pts, patients; RD, residual disease detected by central flow cytometry. * Retrospective risk classification according to Table S1 including genetic and response criteria for the BFM cohort and genetic and RD criteria for the COG cohort (AAML1031 definition). † This interval includes patients from 04/2009 until 07/2013 for the BFM cohort. ‡ This interval includes patients from 08/2013 until 2017 for the BFM cohort.

## Data Availability

A detailed data sharing statement is provided in Supplementary Materials.

## Supplementary Materials

The following are available online at https://www.mdpi.com/article/10.3390/cancers13102336/s1, Data sharing statements; Table S1: Definitions; Table S2: Baseline characteristics BFM cohort; Table S3: Baseline characteristics COG cohort; Table S4: HSCT; Table S5: Additional outcome results BFM cohort; Figure S1: BFM CONSORT Diagram; Figure S2: I-BFM 2001/01 vs. BFM Registry; Figure S3: HSCT; Figure S4: Time from initial diagnosis; Figure S5: Response.
