# Supplementary material for: Survival Following Relapse in Children with Acute Myeloid Leukemia: A Report from AML-BFM and COG
Source: Cancers (Basel). 2021 May 12;13(10):2336. doi: 10.3390/cancers13102336 (PMC8151466; doi:10.3390/cancers13102336)
Supplement: Supplementary file 1 [file cancers-13-02336-s001.zip › sup_rasche.pdf]

Supplementary Material

Data Sharing Statments

BFM – The AML-BFM Study Group Data Sharing policy describes the release and use of AML-BFM individual subject data for use in research projects in accordance with EU-Directive of Good Clinical Practice, the guidelines of the German Research Foundation (DFG) and the German Society of Pediatric Oncology and Hematology (GPOH). Only data expressly released from the oversight of the relevant AML-BFM Data and Safety Monitoring Committee (DSMC) are available to be shared. Data sharing will ordinarily be considered only after the primary study manuscript is accepted for publication. For phase III trials, individual-level de-identified datasets that would be sufficient to reproduce results provided in a publication containing the primary study analysis can be requested from the AML-BFM data management. Data are available to researchers who wish to analyze the data in secondary studies to enhance the public health benefit of the original work and agree to the terms and conditions of use. For non-phase III trials, data are available following the primary publication. An individual-level de-identified dataset containing the variables analyzed in the primary results paper can be expected to be available upon request. Requests for access to AML-BFM protocol research data should be sent to the AML-BFM Study Group offices. Data are available to researchers whose proposed analysis is found by the AML-BFM research board to be feasible and of scientific merit and who agree to the terms and conditions of use.

For all requests, no other study documents, including the protocol, will be made available and no end date exists for requests. In addition to above, release of data collected in a clinical trial conducted under a binding collaborative agreement between AML-BFM Study Group and a pharmaceutical/biotechnology company must comply with the data sharing terms of the binding collaborative/contractual agreement and must receive the proper approvals.

COG – The Children's Oncology Group Data Sharing policy describes the release and use of COG individual subject data for use in research projects in accordance with National Clinical Trials Network (NCTN) Program and NCI Community Oncology Research Program (NCORP) Guidelines. Only data expressly released from the oversight of the relevant COG Data and Safety Monitoring Committee (DSMC) are available to be shared. Data sharing will ordinarily be considered only after the primary study manuscript is accepted for publication. For phase 3 studies, individual-level de-identified datasets that would be sufficient to reproduce results provided in a publication containing the primary study analysis can be requested from the NCTN/NCORP Data Archive at https://nctn-data-archive.nci.nih.gov/. Data are available to researchers who wish to analyze the data in secondary studies to enhance the public health benefit of the original work and agree to the terms and conditions of use. For non-phase 3 studies, data are available following the primary publication. An individual-level de-identified dataset containing the variables analyzed in the primary results paper can be expected to be available upon request. Requests for access to COG protocol research data should be sent to: datarequest@childrensoncologygroup.org. Data are available to researchers whose proposed analysis is found by COG to be feasible and of scientific merit and who agree to the terms and conditions of use.

For all requests, no other study documents, including the protocol, will be made available and no end date exists for requests. In addition to above, release of data collected in a clinical trial conducted under a binding collaborative agreement between COG or the NCI Cancer Therapy Evaluation Program (CTEP) and a pharmaceutical/biotechnology company must comply with the data sharing terms of the binding collaborative/contractual agreement and must receive the proper approvals.

Supplemental Table S1: Definitions

	AML-BFM*	COG
**Initial risk stratification**		
**Standard risk**	• Inv(16)(p13.1q22), t(16;16)(p13;q22)<br>• t(8;21)(q22;q22)<br>• t(1;11) (q21;q23)<br>• Normal karyotype and NPM1-mutation<br>• Normal karyotype and CEBPA (double mutation)	Low risk mutation regardless of monosomy 7, monosomy 5, or del5q and regardless of RD at end of Induction I:<br>• Inv(16)(p13.1q22), t(16;16)(p13;q22)<br>• t(8;21)(q22;q22);<br>• NPM or CEBPα
**Intermediate risk**	All patients with de-novo AML, who do not belong to the standard-risk group (favorable prognosis) or to the high risk group (unfavorable prognosis)<br>• Patients with SR and FLT3/ITD	All patients with de-novo AML, without favorable or unfavorable prognostic genetic features
**High risk**	• Abnormalities in chromosome 12p/ t(2;12);<br>• monosomy 5/5q-;<br>• WT1mut and FLT3-ITD<br>• monosomy 7 (not in combination with favorable/MLL- aberrations)<br>• t(4;11)(q21;q23); KMT2A/AF4;<br>• t(5;11)(q35.3;p15); NUP98/NSD1;<br>• t(6;11)(q27;q23)); KMT2A/AF6;<br>• t(10;11)(p12;q23); KMT2A/AF10;<br>• t(6;9)(p23;q34)<br>• t(7;12)(q36;p13)<br>• t(9;22)(q34;q11)<br>• complex karyotype (three or more aberrations, including at least one structural aberration, without favorable genetics and without KMT2A-rearrangement.)<br>• inv(3)(q21q26.2)/t(3;3)(q21;q26.2)	• FLT3/ITD+ with high allelic ratio > 0.4 regardless of low risk features.<br>• Presence of monosomy 7, monosomy 5, or del5q<br>• Evidence of residual AML (RD ≥ 0.1%) at end of Induction I (AAML1031 only)
**Re-stratification**	Therapy response (evaluated via morphology and immunophenotyping) after the 1st (~ day 28) and 2nd (~ day 56) induction was used for a subsequent re-stratification. Re-stratification into the intermediate or high risk group is based on nonresponse (≥10% blasts after 1st or ≥5% after 2nd induction).	Not applicable
**First Relapse**		
**Relapse**	Reappearance of leukemic blasts in the peripheral blood, re-infiltration of BM with ≥5% distinct blasts (in case of questionable results control after 2–3 weeks) not to be assigned to any other cause, or distinctive leukemic infiltration elsewhere following CR or partial remission lasting at least 4 weeks. Reappearance or development of cytologically proven extramedullary disease was considered as relapse.	≥ 5% blasts in the bone marrow or relapse at extramedullary sites not attributable to any other cause (e.g., bone marrow regeneration) after documented CR at end of Induction II.
**Response**		
**Early treatment response after first re-induction**		
**Good response**	≤ 20% leukemic blasts in the BM	Not applicable
**Poor response**	More than 20% leukemic blasts	Not applicable
**Response after up to two courses of re-induction therapy**		
**Complete remission (CR)**	< 5% leukemic blasts in the BM with signs of normal hematopoiesis and with clear signs of regeneration of normal peripheral-blood cell production (platelets ≥ 80 × 109/L without transfusions, neutrophils ≥ 1.0 × 10^9/L), and furthermore no leukemic cells in the peripheral blood or anywhere else	Not applicable
**Complete remission with partial regeneration (CRp)**	< 5% leukemic blasts in the BM with signs of normal hematopoiesis and with clear signs of regeneration of normal peripheral-blood cell production (platelets ≥ 50 × 10^9/L without transfusions, neutrophils ≥ 0.5 × 10^9/L), and no leukemic cells in the peripheral blood or anywhere else.	Not applicable
**Complete remission with incomplete recovery (CRi)**	< 5% leukemic blasts in the BM with minimal signs of regeneration of normal peripheral-blood cell production (platelets ≥ 20 × 10^9/L without transfusions and neutrophils ≥ 0.5 × 10^9/L), and no leukemic cells in the peripheral blood or anywhere else.	Not applicable
**Aplasia**	< 5% leukemic blasts in the BM in an aplastic BM with no signs of peripheral blood count recovery (platelets < 20 × 10^9/L without transfusions or neutrophils < 0.5 × 10^9/L).	Not applicable
**Nonresponse**	≥ 5% leukemic blasts in the BM and/or documented leukemic cells elsewhere after two complete courses of chemotherapy.	

* Patients of the AML-BFM study group have been categorized according to the risk group definition of AML-BFM study 2012. It was used prospectively in AML-BFM registry 2012 and study 2012, while previous patients have been analyzed retrospectively for this purpose.

Supplemental Table S2: Baseline characteristics BFM cohort

		First Relapse	First Relapse DNX-FLA	Other	p(chi)-value
**Number of patients (%)**		197 (100%)	156 (100%)	41 (100%	
**Initial characteristics**					
**Age (years), median (range)**		9.4 (0.1 – 18.0)	9.5 (0.2 – 17.9)	9.1 (0.1 – 18.0)	0.904
**Gender**	Male	108 (55%)	88 (56%)	20 (49%)	0.3824
	Female	89 (45%)	68 (44%)	21 (51%)	
**FAB**	M0	12 (6%)	8 (5%)	4 (10%)	0.0216
	M1/M2	73 (37%)	61 (39%)	12 (29%)	
	M4/M5	89 (45%)	72 (46%)	17 (41%)	
	M4eo	9 (5%)	7 (5%)	2 (5%)	
	M6	1 (<1%)	1 (<1%)	---	
	M7	9 (5%)	6 (4%)	3 (7%)	
	Non-classified	4 (2%)	1 (0.6%)	3 (7%)	
**Blood counts**	WBC (x10^3/dl) median (range)	23.9 (0.4 – 484.3)	24.0 (1.1 – 384.0)	20.06 (0.4 – 484.3)	0.4662
**Risk group**	Standard	31 (17%)	29 (21%)	2 (5%)	0.0031
	Intermediate	81 (45%)	67 (48%)	14 (36%)	
	High	68 (38%)	45 (32%)	23 (59%)	
	No data	17	15	2	
**Initial response**	CR	185 (94%)	151 (97%)	34 (83%)	0.0010
**Previous treatment regimen**					
**Initial treatment protocol**	AML-BFM study 2004	127 (65%)	101 (65%)	26 (63%)	0.5405
	AML-BFM registry 2012	59 (30%)	45 (29%)	14 (34%)	
	AML-BFM study 2012	11 (6%)	10 (6%)	1 (2%)	
**HSCT**	HSCT at initial disease	28 (14%)	15 (10%)	13 (32%)	0.0002
**Relapse characteristics**					
**Age**	At first relapse (years), median (range)	10.5 (0.5 – 21.0)	10.7 (0.7 – 20.9)	9.7 (0.5 – 21.0)	0.8777
**Time to subsequent relapse**	Early 1st relapse	91 (46%)	24 (59%)	67 (43%)	0.0748
	Late 1st relapse	106 (54%)	17 (41%)	89 (57%)	
**Early death**		11 (6%)	6 (4%)	5 (12%)	0.0383

Table legend: Abbreviations: HSCT, hematopoietic stem cell transplantation; WBC, white blood cell; count CR; complete remission.

Supplemental Table S3: Baseline characteristics COG cohort

		First relapse
**Number of patients (%)**		852 (100%)
**Initial characteristics**		
**Age (years) at relapse, median (range)**		9.8 (0.33 – 32.5)
**Classification**	AML with t(8;21)(q22;q22); RUNX1-RUNX1T1	63 (7.4%)
	AML with inv(16)(p13q22) or t(16;16)(p13;q22); CBFB-MYH11	65 (7.6%)
	AML with mutated NPM1	10 (1.2%)
	AML with mutated CEBPA	14 (1.6%)
	AML with t(6;9)(p23;q34); DEK-NUP214	3 (0.4%)
	AML with 11q23 (MLL) abnormalities	137 (16.1%)
	AMKL with t(1;22)(p13;q13); RMB15-MKL1	6 (0.7%)
	Others	554 (65.0%)
**Gender**	Male	465 (54.6%)
	Female	387 (45.4%)
**Risk groups according to AAML1031**	Low	608 (74.1%)
	High	237 (27.8%)
	Unknown	7 (0.8%)
**Initial response**	RD+	222 (26.0%)
	RD-	543 (63.7%)
	Unevaluable	87 (10.2%)
**Previous treatment regimen**		
**Initial Therapy**	AAML0531	358 (42.0%)
	AAML1031	494 (58.0%)
**Prior HSCT**	No	670 (78.6%)
	Yes	117 (13.7%)
	Unknown	65 (7.6%)
**Relapse characteristics**		
**Relapse Time Period**	2007-2009	203 (23.8%)
	2010-2013	297 (34.9%)
	2014-2017	333 (39.1%)
	2018-2018	19 (2.2%)
**Time to subsequent relapse**	Early first relapse	500 (58.7%)
	Late first relapse	352 (41.3%)

Table legend: Abbreviations: HSCT, hematopoietic stem cell transplantation; RD: residual disease.

Supplemental Table S4: HSCT

p(chi)=0.0286	No HSCT after first relapse		HSCT after first relapse	
	**N**	**%**	**N**	**%**
**Period**				
**Relapse year interval 04/01until 03/05***	80	31	180	69
**Relapse year interval 4/05 until 03/09***	57	24	181	76
**Relapse year interval 04/09 until 07/13****	22	19	96	81
**Relapse year interval 08/13 until 12/17****	14	18	62	82

Table legend: HSCT, hematopoietic stem cell transplantation. * Patients of study I-BFM Relapse 2001/01. **Patients of the current BFM cohort.

Supplemental Table S5: Additional outcome results BFM cohort

	Patients		5-year pOS		p (Log rank)*
	n	%	%	SE	
**Total no. of patients with first relapse**	**203**	**100**	**42**	**4**	
**All patients with sufficient data**	**197**	**100**	**42**	**4**	
• Nonresponse at initial disease	12	6	0	0	0.031
• Complete remission at initial disease	185	94	45	4	
• Initial treatment: AML-BFM 04	127	65	49	7	0.32
• Initial treatment: AML-BFM Registry 12 + Study 12	70	30	39	5	
• Relapse treatment: DNX-FLA(G) +/- FLA(G)	156	79	44	4	0.20
• Relapse treatment: Others	41	21	36	8	
**Response data after first DNX-FLA(G) available**	**140**	**100**			
• Good response after DNX-FLA(G) (≤ 20% leukemic blasts) after first re-induction	122	87	49	5	0.058
• Poor response after DNX-FLA(G) (> 20% leukemic blasts) after first re-induction	18	13	16	13	

Table legend: SE, standard error; pOS, probability of overall survival; CI ED, cumulative incidence of early death. DNX-FLA(G), liposomal daunorubicin, fludarabine, cytarabine with or without granulocyte-colony-stimulating factor.

Supplemental Figure S1: BFM CONSORT Diagram

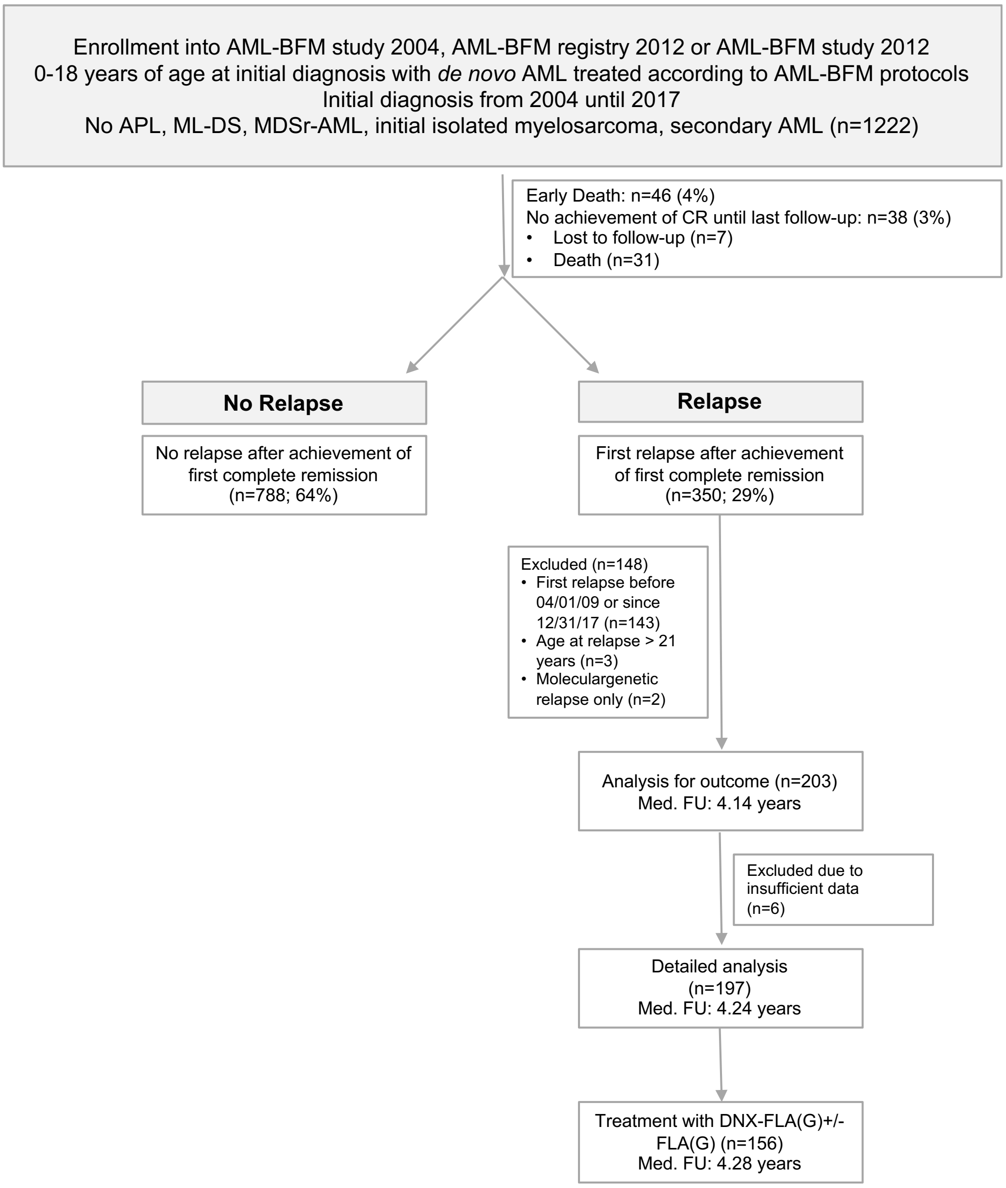

CONSORT flow diagram showing patients of the AML-BFM studies and registries from 2004 und 2017 that have been included or excluded from the retrospective analysis. ML-DS, patients with Down syndrome myeloid leukemia; APL, acute promyeloblastic leukemia; MDSr-AML, AML with myelodysplasia related changes; FU, follow-up; DNX-FLA(G), liposomal daunorubicin, fludarabine, cytarabine with or without granulocyte-colony-stimulating factor.

Supplemental Figure S2: I-BFM 2001/01 vs. BFM Registry

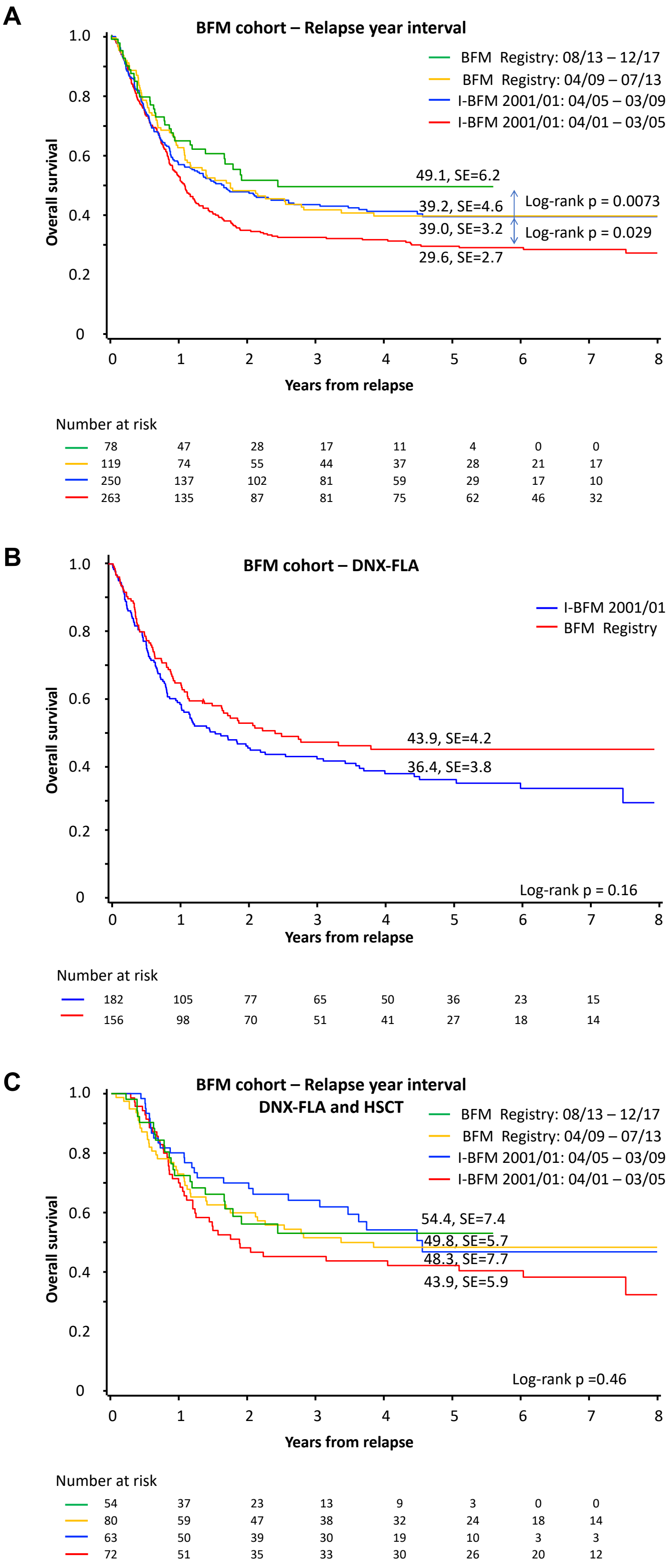

(A) 5-year overall survival in patients with pediatric AML with diagnosed first relapse from 04/09 until 07/13 and 08/13 – 12/17 in the AML-BFM Registry or from 04/01 until 03/05 and 04/05 – 03/09 in I-BFM Relapse 2001/01. (B) 5-year overall survival of patients in the BFM registry or I-BFM Relapse 2001/01 treated with DNX-FLA. (C) 5-year overall survival in patients treated with DNX-FLA and HSCT from 04/09 until 07/13 and 08/13 – 12/17 in the AML-BFM Registry or from 04/01 until 03/05 and 04/05 – 03/09 in I-BFM Relapse 2001/01.

Supplemental Figure S3: HSCT

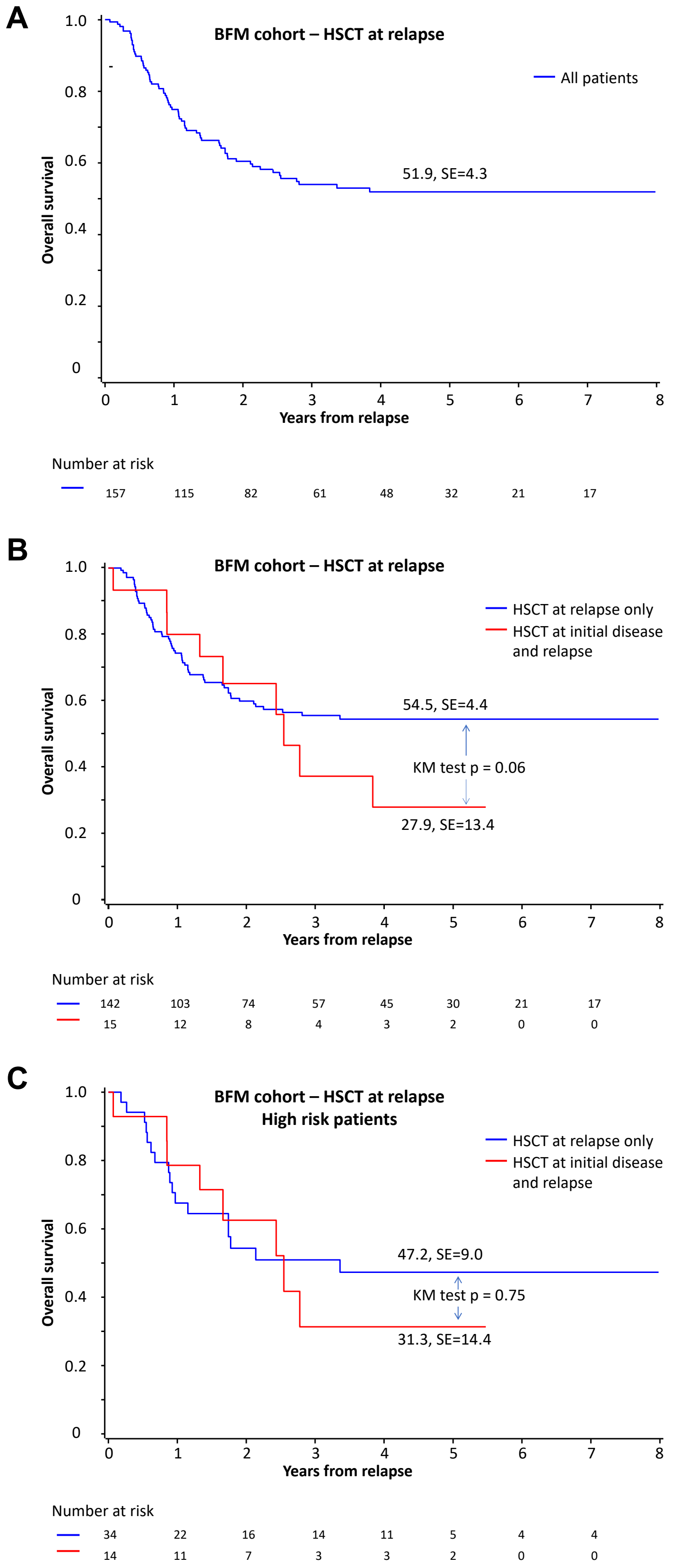

(A) 5-year overall survival in all patients of the BFM registry receiving a hematopoietic stem cell transplantation (HSCT) after first relapse. (B) 5-year overall survival in transplanted patients with or without a prior HSCT at initial disease. (C) 5-year overall survival in transplanted patients with or without a prior HSCT at initial disease limited to patients with high risk stratification at initial disease.

Supplemental Figure S4: Time from initial diagnosis

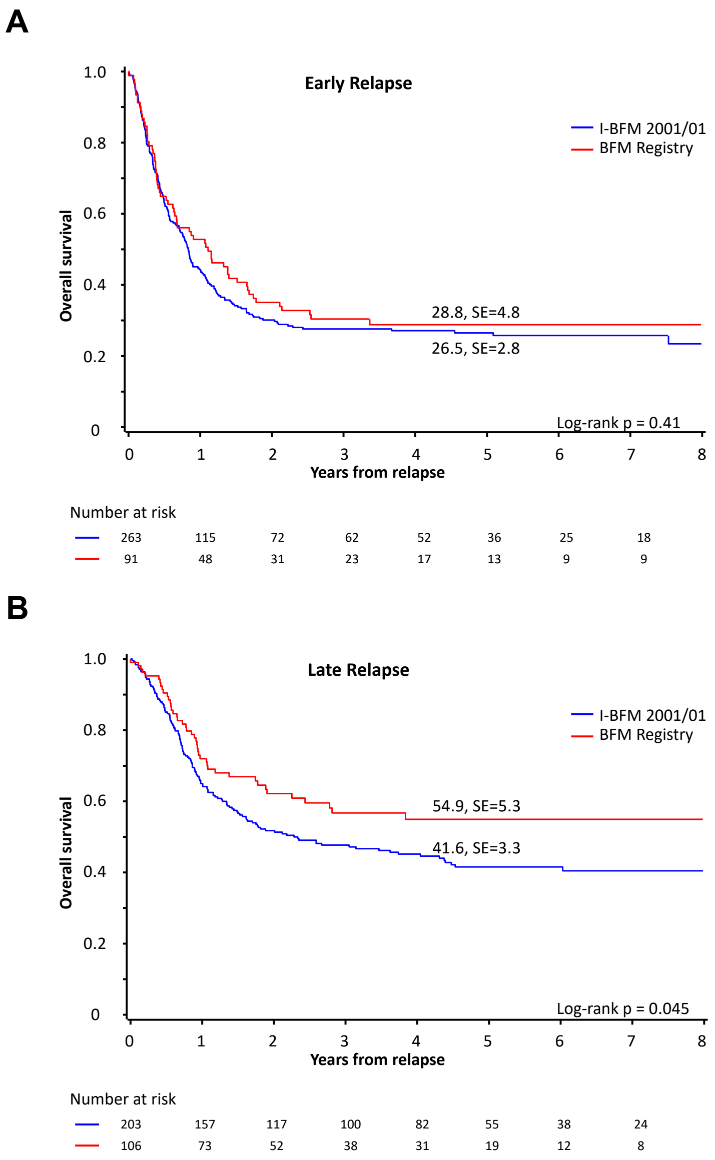

(A) 5-year overall survival of patients in the BFM registry or I-BFM Relapse 2001/01 with early relapse. (B) 5-year overall survival of patients in the BFM registry or I-BFM Relapse 2001/01 with late relapse defined as relapse within or after one year of diagnosis.

Supplemental Figure S5: Response

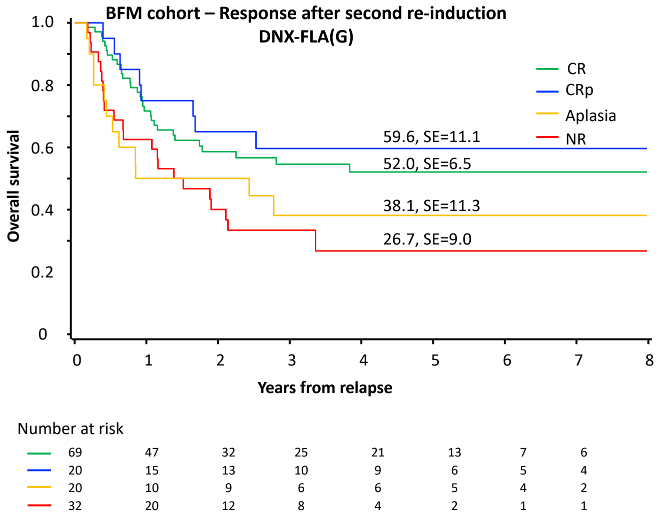

5-year overall survival in patients with pediatric AML with first relapse based on the detailed response to DNX-FLA(G) +/- FLA(G). Patients with Cri (n=6) are excluded due to small patient numbers.
